# Krebs von den Lungen 6 (KL-6) as a marker for disease severity and persistent radiological abnormalities following COVID-19 infection at 12 weeks

**DOI:** 10.1371/journal.pone.0249607

**Published:** 2021-04-29

**Authors:** David T. Arnold, Charmaine Donald, Max Lyon, Fergus W. Hamilton, Anna J. Morley, Marie Attwood, Alexandra Dipper, Shaney L. Barratt

**Affiliations:** 1 Academic Respiratory Unit, North Bristol NHS Trust, Bristol, United Kingdom; 2 Department of Immunology, North Bristol NHS Trust, Bristol, United Kingdom; 3 Bristol Interstitial Lung Disease Service, North Bristol NHS Trust, Bristol, United Kingdom; 4 Bristol Centre for Antimicrobial Research (BCARE), North Bristol NHS Trust, Bristol, United Kingdom; Myongji Hospital, Hanyang University College of Medicine, REPUBLIC OF KOREA

## Abstract

**Introduction:**

Acute presentations of COVID-19 infection vary, ranging from asymptomatic carriage through to severe clinical manifestations including acute respiratory distress syndrome (ARDS). Longer term sequelae of COVID-19 infection includes lung fibrosis in a proportion of patients. Krebs von den Lungen 6 (KL-6) is a mucin like glycoprotein that has been proposed as a marker of pulmonary epithelial cell injury. We sought to determine whether KL-6 was a marker of 1) the severity of acute COVID-19 infection, or 2) the persistence of symptoms/radiological abnormalities at medium term follow up.

**Methods:**

Prospective single centre observational study.

**Results:**

Convalescent KL-6 levels were available for 93 patients (male 63%, mean age 55.8 years) who attended an 12-week follow up appointment after being admitted to hospital with COVID-19. For 67 patients a baseline KL-6 result was available for comparison. There was no significant correlations between baseline KL-6 and the admission CXR severity score or clinical severity NEWS score. Furthermore, there was no significant difference in the baseline KL-6 level and an initial requirement for oxygen on admission or the severity of acute infection as measured at 28 days. There was no significant difference in the 12-week KL-6 level and the presence or absence of subjective breathlessness but patients with abnormal CT scans at 12 weeks had significantly higher convalescent KL-6 levels compared to the remainder of the cohort (median 1101 IU/ml vs 409 IU/ml).

**Conclusions:**

The association between high KL-6 levels at 12 weeks and persisting CT abnormalities (GGO/fibrosis), is a finding that requires further exploration. Whether KL-6 may help differentiate those patients with persisting dyspnoea due to complications rather than deconditioning or dysfunctional breathing alone, is an important future research question.

## Introduction

Acute presentations of COVID-19 infection vary, ranging from asymptomatic carriage through to severe clinical manifestations including acute respiratory distress syndrome (ARDS) [1–3]. As we emerged from the first wave of the coronavirus pandemic, new understanding of the potential longer term sequelae of COVID-19 infection transpired, including the persistence of breathlessness and fatigue several months after the initial acute infection, with a proportion of patients having evidence of lung fibrosis [4, 5].

Krebs von den Lungen 6 (KL-6) is a mucin like glycoprotein distributed mainly on the surface of type II alveolar epithelial cells (AECs) and respiratory bronchiolar epithelial cells within the normal lung [6], that has been shown to exert chemotactic and anti-apoptotic effects on fibroblast cells [7]. It has been proposed as a marker of pulmonary epithelial cell injury; high levels of KL-6 have been demonstrated in the epithelial lining fluid (ELF) of patients with acute lung injury (ALI) and interstitial lung disease compared to controls [8, 9], with significantly elevated plasma levels of KL-6 in non-survivors of ALI compared to survivors. Elevated serum KL-6 levels have been correlated with the severity of IPF [10] and ILD associated with connective tissue disease [11–14] and may also be a useful predictor of early progression in patients with systemic sclerosis associated ILD [11–13]. High circulating levels of KL-6 have additionally been reported in *Pneumocystis jivorecii* and viral respiratory infection [15, 16].

Clinical phenotyping of COVID-19 patients at admission, identifying those at risk of developing ARDS/severe disease, or predicting future outcomes within this heterogeneous population, is the first step to a personalised approach in management of this condition.

The specific purpose of this study was to explore:

1. the relationship between baseline KL-6 levels and the severity of acute COVID-19 infection.
2. the relationship between 12 week KL-6 levels and the persistence of symptoms or radiological abnormalities at 12 weeks.

## Methods

### Study participants

All adults were participants of the single centre, prospective, observational Diagnostic and Severity markers of COVID-19 to Enable Rapid triage study (DISCOVER). This study recruited consecutive adults admitted to North Bristol NHS trust, a large secondary care hospital in the South-West of England, with COVID-19 (between 30 March and 3 June 2020) as previously described [4]. The inclusion criteria were adult patients (>18 years old) with typical symptoms of COVID-19 (e.g. respiratory illness with cough and breathlessness) and a positive PCR result for SARS-CoV-2 or a clinico-radiological diagnosis of COVID-19, namely presenting with typical symptoms, compatible chest X-ray findings and alternative causes excluded or considered unlikely. The study received approval by South Yorkshire Research Ethics Committee 20/YH/0121, NIHR CRN approval number: 45469).

### Assessments

Baseline demographics, clinical information including the National Early Warning Score (NEWS) and blood test results were extracted from the medical record. NEWS is a clinical illness severity score incorporating respiratory rate, oxygen saturation, use of supplemental oxygen, temperature, systolic blood pressure, heart rate, and consciousness level [17].

Radiological severity scores of baseline chest radiographs (CXR) were calculated as previously described [4, 18]. Briefly, a score of 0–4 was assigned by a respiratory physician or infectious diseases physician to each lung depending on the extent of abnormality; 0 = no involvement, 1 = <25%, 2 = 25–49%, 3 = 50–75%, 4 = >75% involvement. The nature of the abnormality 1) consolidation, 2) ground-glass opacity (GGO), 3) nodular opacity, and 4) reticular opacity 5) atelectasis 6) pleural pathology were evaluated according to standardized terminology [19].

All patients were remotely followed up at 28 days to obtain information on short term outcomes including mortality, hospital length of stay, intensive care admission alongside need for renal replacement therapy, inotropic support or ventilation. At 28 days, survivors were categorised as having severe disease (if received invasive mechanical ventilation (IMV), non-invasive ventilation (NIV) and/or had an intensive care admission) during their admission, moderate disease (received supplementary oxygen during admission) or mild disease (no supplementary oxygen or intensive care admission).

All patients were followed up by face to face consultation at approximately 12 weeks with clinical assessment, repeat blood tests, CXR, and spirometry by a respiratory physician or infectious disease consultant.

### Krebs-von den Lungen (KL-6) assay

Serum KL-6 levels were measured in blood samples obtained at the participant’s 12 week follow-up consultation. Where available, paired stored serum samples, in excess to diagnostic requirements and originally taken at hospital admission were also tested. KL-6 levels (IU/ml) were determined using a chemiluminescence assay “Lodicules^®^ G KL-6” (Fujirebio Europe, UK) according to the manufacturer’s instructions. The results were measured using an automated immunoassay system (LUMIPULSE G1200; Fujirebio, Inc., Tokyo, Japan).

Serum KL-6 levels were also tested in randomly selected, archived and de-identified serum samples of patients with a multidisciplinary team consensus diagnosis of idiopathic pulmonary fibrosis (n = 20), which acted as positive control for lung fibrosis (REC number 17/SW/0227) and n = 20 serum samples of patients with heart failure to act as negative controls for lung fibrosis (REC number 08/H0102/11).

### Statistical analysis

Categorical variables are reported as absolute numbers and percentages. Normality of continuous data was initially verified using D’Agostino and Pearson normality test. Mean and standard deviation (SD) were used to describe parametric data; median and interquartile range (IQR) for non-parametric data. Differences among two groups were verified by t-test with Welch’s correction for parametric data and Mann-Whitney U for non-parametric data. χ^2^-tests were used for categorical data. Kruskal-Wallis was used for comparison of multiple non-parametric groups. Spearman’s rank was used to determine statistically significant correlations between variables. Data were analysed using GraphPad Prism version 8.0. A P value of <0.05 was considered statistically significant.

## Results

Patients were followed-up at a median of 83 days (IQR 74–88 days) after hospital admission and 90 days (IQR 80–97 days) after COVID-19 symptom onset.

Convalescent KL-6 levels were available for 93 patients who attended a 12-week follow up appointment after being admitted to hospital with COVID-19 (male gender 63.4%, mean age 54.8 years). Baseline demographics for these patients are shown in Table 1. The vast majority of the cohort (77/93; 83%) were found to be SARS-COV2 antibody positive at 12 weeks. For 67 patients an admission KL-6 result was available for comparison.

**Table 1:** Baseline demographics of patients with 12-week serum Krebs von den Lungen-6 (KL-6) levels.

Demographic	Results (n = 93)
Male n, (%)	59 (63.4)
Mean age years, (SD)	54.8 (14.5)
Severity of infection (at 28 days)	
Mild, n (%)	22 (23.7)
Moderate, n (%)	54 (58.0)
Severe, n (%)	17 (18.0)
PCR positive during acute infection, n (%)	74 (79.6)
Antibody positivity at 12 weeks, n (%)	77 (82.8)
Median baseline KL-6 IU/ml (IQR)	365 (233–493), n = 67
Median 12-week KL-6 IU/ml (IQR)	412 (283–613)
**Spirometry (reported as mean and SD) at 12 weeks**	
FEV1 (litres)	2.78 (0.91)
FEV1% predicted	89.7 (16.6)
FVC (litres)	3.52 (1.12)
FVC % predicted	90.4 (15.1)
FEV1/FVC ratio	78.2 (11.5)

Abbreviations: PCR, polymerase chain reaction; n, number; %, percentage; IQR, interquartile range; SD, standard deviation.

**Fig 1:**
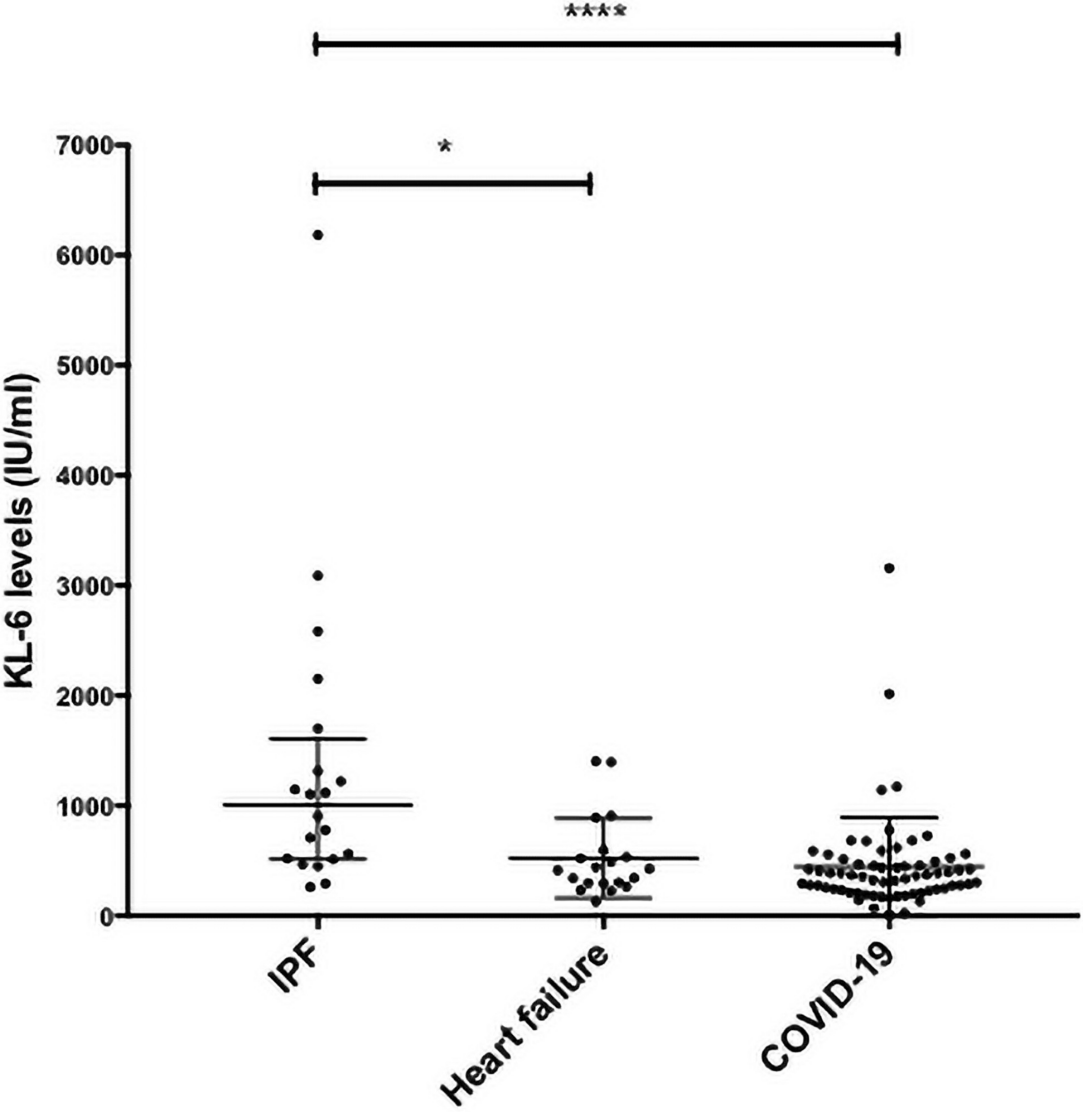
Serum Krebs von den Lungen (KL-6) levels in COVID-19 patients on admission compared to populations of patients with Idiopathic Pulmonary Fibrosis (IPF) and heart failure. Baseline KL-6 levels in patients with COVID-19 were comparable to those measured in a cohort of heart failure patients but statistically lower than serum levels measured in a mild to moderate cohort of IPF patients (baseline KL-6 COVID-19 365IU/ml (IQR 233–493) n = 67, heart failure 419 IU/ml (IQR 294–581) n = 20, IPF 1005 (IQR 514–1604 IU/ml) n = 20, Kruskal-Wallis * p<0.05, ****p<0.0001).

Baseline KL-6 levels in patients with COVID-19 were comparable to those measured in a cohort of heart failure patients but statistically lower than serum levels measured in a mild to moderate cohort of IPF patients (baseline KL-6 COVID-19 365IU/ml (IQR 233–493) n = 67, heart failure 419 IU/ml (IQR 294–581) n = 20, IPF 1005 (IQR 514–1604 IU/ml) n = 20, Kruskal-Wallis, p<0.0001) (Fig 1).

### Does baseline KL-6 predict the severity of acute COVID infection?

There were no significant correlations between baseline KL-6 and the admission CXR severity score (Spearman’s p = 0.06) or NEWS score (Spearman’s p = 0.112). Furthermore, there was no significant difference in the baseline KL-6 level and an initial requirement for oxygen on admission; KL-6 levels in those requiring oxygen (saturations <94% air) 397IU/ml (IQR 256–583) (n = 28) vs those not requiring oxygen (saturations >94% air) 302IU/ml (210–448) (n = 39), p = 0.393.

**Fig 2:**
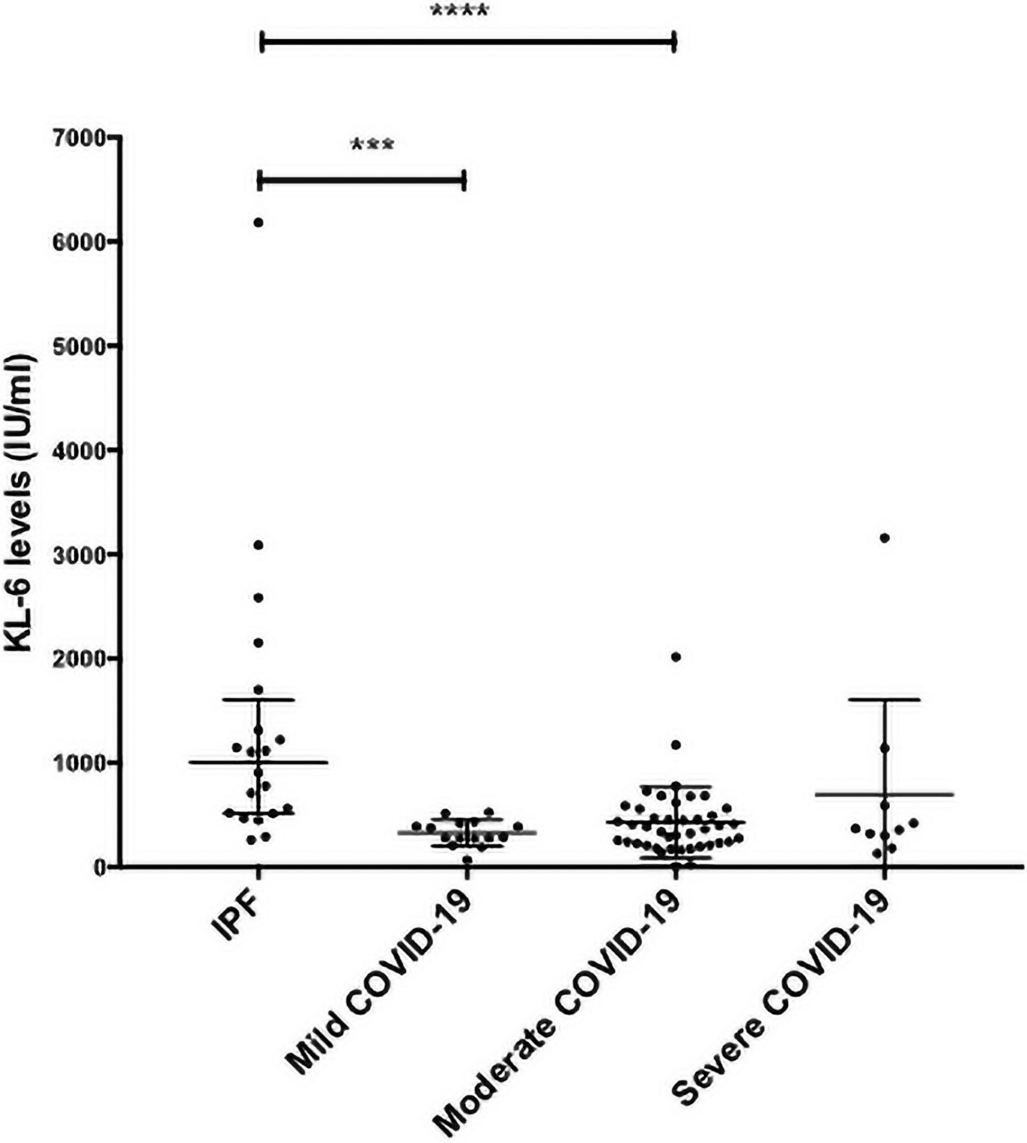
Serum Krebs von den Lungen (KL-6) levels in COVID-19 patients according to disease severity at 28 days following admission and compared to a population of patients with Idiopathic Pulmonary Fibrosis (IPF). There was no statistical difference in the baseline KL-6 and the severity of the acute infection as measured at 28 days (Kruskal-Wallis, p>0.05). KL-6 levels in patients with mild-moderate IPF were statistically higher than those with mild (*** p<0.001) or moderate COVID-19 (****p<0.0001).

There was no statistical difference in the baseline KL-6 and the severity of the acute infection as measured at 28 days (Kruskal-Wallis, p>0.05) (Fig 2).

### Is KL-6 a marker of symptoms, spirometry or radiology at 12 weeks?

At follow up, 36% (24/67) patients described ongoing symptoms of breathlessness. There was no significant difference in the 12-week KL-6 level and the presence or absence of subjective breathlessness (breathlessness 425IU/ml (IQR 347–549) vs no breathlessness 422IU/ml (IQR 282–618), p = 0.583, Mann-Whitney U). Similarly, KL-6 levels at 12-weeks did not differ significantly between those with MRC scores 0–1 compared to those scoring an MRC of 2 or more (363 IU/ml (IQR 270–628), n = 35 versus 454 IU/ml (IQR 348–677), n = 32 p = 0.185, Mann Whitney U).

**Fig 3:**
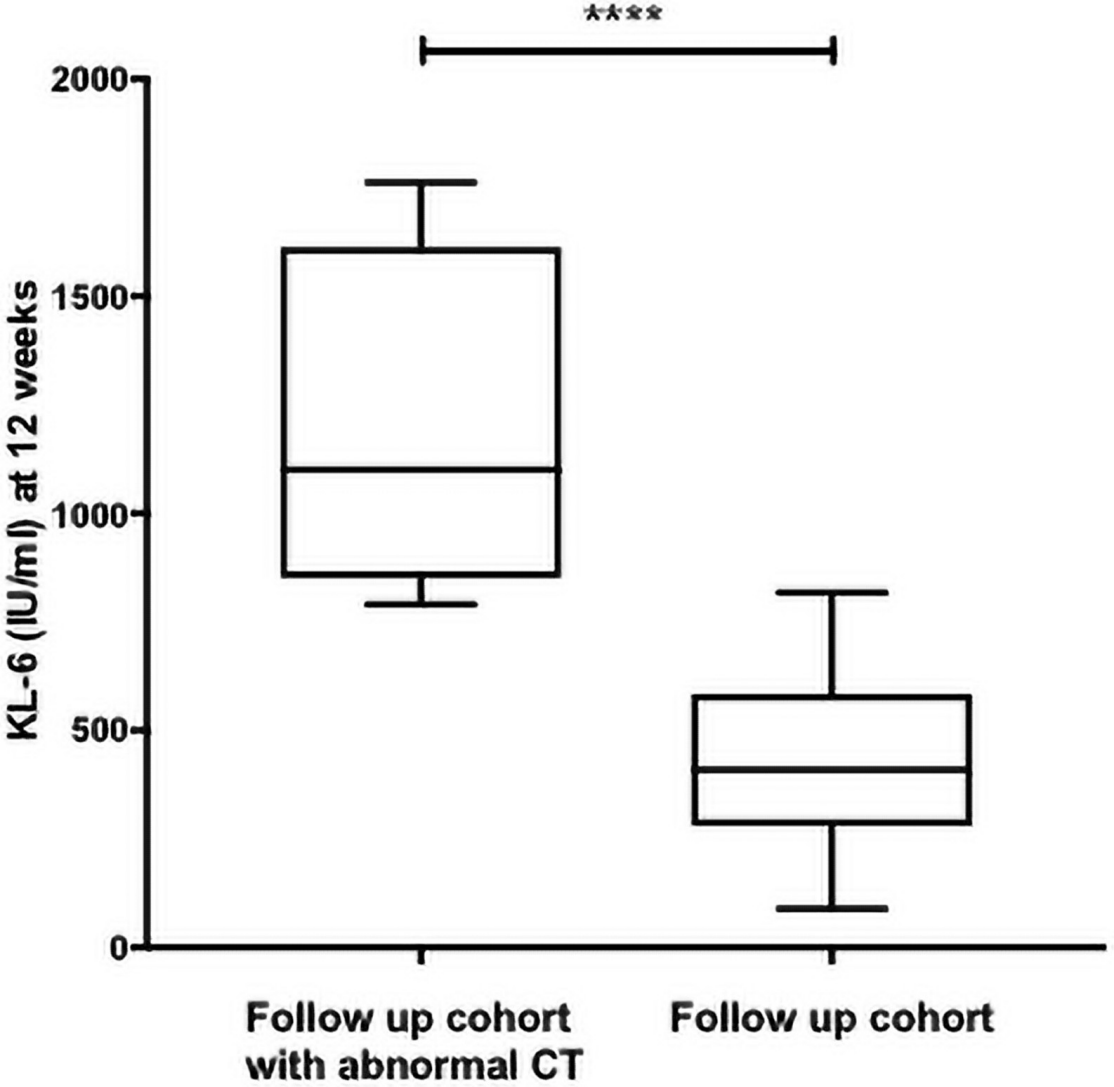
Serum Krebs von den lungen (KL-6) levels in patients with abnormal CT scans at 12 weeks. Patients with abnormal CT scans at 12 weeks had significantly higher convalescent KL-6 levels compared to the remainder of the cohort (median KL-6 1101 IU/ml, n = 4 vs 409 IU/ml, Mann Whitney U, p<0.0001****).

We have previously described the radiological outcomes and spirometry results of the entire DISCOVER prospective cohort at 12 weeks. [4] In this smaller subgroup of patients with paired baseline and 12 week KL-6 levels, there was a statistically significant but weak negative correlation between 12-week KL-6 levels and FVC% predicted (Spearman’s rank, p = 0.014, r = -0.258). A minority of patients (11/66, 17%) had an abnormal chest X-ray at 12 weeks (one patient declined follow-up CXR), prompting a clinical decision to perform High Resolution Computed Tomography Chest (HRCT) in n = 4. Two HRCTs showed bilateral ground glass opacification and 2 showed evidence of established pulmonary fibrosis. Patients with abnormal CT scans at 12 weeks had significantly higher convalescent KL-6 levels compared to the remainder of the cohort (median KL-6 1101 IU/ml vs 409 IU/ml) (Fig 3).

Three patients (3/67) had 12 week KL-6 levels that were >1000IU/ml; the cases are briefly described below:

Case 1: 64 year old male with severe COVID-19 disease, requiring IMV. KL-6 levels had improved compared to baseline levels of >3000 IU/ml. CXR at follow up had significantly improved compared to admission but remained abnormal. Contemporaneous HRCT at follow up demonstrated bilateral ground glass opacification.
Case 2: 57 year old male with moderate COVID-19 disease. Levels of KL-6 at follow-up had risen compared to baseline levels (778 rising to 1165IU/ml). CXR had improved compared to admission with persistent left sided parenchymal change. HRCT scan demonstrated ground glass opacification.
Case 3: 71 year old male with moderate COVID-19 disease. Levels of KL-6 at follow up had risen compared to baseline levels (240 rising to 1036 IU/ml). Follow up CXR showed bilateral parenchymal abnormalities. HRCT demonstrated established fibrosis, likely to predate the COVID-19 illness with superimposed consolidation/ground glass opacification.

## Conclusion

Severe manifestations of COVID-19 include pneumonia and ARDS that may require intensive care admission and ventilatory support. Pulmonary fibrosis is a recognised sequelae of ARDS. An increasing number of studies have shown that longer term consequences of COVID-19 infection includes pulmonary fibrosis in a subset of patients [4, 5], with the potential for persistent or even progressive disease [5]. Clinicians currently lack tools to accurately predict both short term outcomes of COVID-19 infection and the possibility of longer term sequelae.

To our knowledge this is the largest prospective study of KL-6 levels in patients with acute COVID-19 infection and one of the first to study convalescent trends in detail with correlation to medium term clinical outcomes. In this cohort, baseline KL-6 were significantly lower than those with confirmed mild to moderate IPF; the archetypal fibrotic lung disease. KL-6 did not predict severity of disease at 28 days, nor the presence of persistent symptoms at 12 weeks.

Existing literature examining a possible role for KL-6 in predicting disease outcomes from COVID-19 infection is limited, has been derived from small scale studies and is potentially conflicting. Differences in subgroup definitions hinder direct comparisons between studies. A prospective study of 22 patients with COVID-19 infection and radiologically confirmed pneumonia [20] showed that KL-6 levels were significantly higher in patients who were mechanically ventilated (severe group, n = 9) compared to those who received pharmacological and oxygen supplementation or non-invasive ventilation (mild to moderate, n = 12) [20]. Xue et al. [21], similarly showed that KL-6 levels were higher in patients with severe disease compared to those with mild COVID-19 disease (mild n = 30, severe n = 33). Patients were classified according to Chinese national healthcare commission (NHC) guidance [22], defining mild COVID as those with mild clinical symptoms, not warranting oxygen therapy and without radiological pneumonia, whilst severe was defined as those patients with pneumonia requiring respiratory support (high flow nasal oxygen, NIV or IMV). In contrast, Frix et al. did not show any correlation between KL-6 levels and admission to intensive care or mortality, but did show that high KL-6 levels were more indicative of severe lung disease based on the admission oxygen saturation levels in ambient air [23].

Our data raised the possibility that high levels of KL-6 at 12 weeks (particularly those >1000IU/ml) may be associated with the presence of persisting lung parenchymal abnormalities at 12 weeks. Alessandro et al. have also recently reported the persistence of high KL-6 levels in a smaller cohort of patients with fibrotic sequelae of COVID-19 longitudinally followed to 9 months [24]. Several other groups have described that dynamic change in serum KL-6 levels appears to reflect disease patterns and may be a marker of therapeutic efficacy or disease progression in other fibrotic interstitial lung diseases [7, 25, 26], thus implicating KL-6 as a potential biomarker for longer term sequelae of COVID-19.

The limitations of this study are recognised. We measured KL-6 at baseline and 12 weeks and as such may have missed dynamic changes and peak KL-6 levels during the intervening period [27]. Furthermore, whilst all but one patient had a follow up CXR at 12 weeks in this prospective cohort, a minority of CT scans were performed. It is widely recognised that CXR has inadequate diagnostic sensitivity and specificity for interstitial lung disease and this limits the conclusions that can be drawn. Large scale, multicentre cohorts such as PHOSP-COVID (www.phosp.org), will be essential to answer this important question.

In conclusion, the association between high KL-6 levels at 12 weeks with persisting CT abnormalities (GGO/fibrosis), is a finding that requires further exploration to determine whether KL-6 may help differentiate those patients with persisting dyspnoea due to complications rather than deconditioning or dysfunctional breathing alone.
